# Effects of Bread Making and Wheat Germ Addition on the Natural Deoxynivalenol Content in Bread

**DOI:** 10.3390/toxins6010394

**Published:** 2014-01-21

**Authors:** Isabel Giménez, Jesús Blesa, Marta Herrera, Agustín Ariño

**Affiliations:** 1 Veterinary Faculty, University of Zaragoza, Zaragoza 50013, Spain; E-Mails: gimenezi@unizar.es (I.G.); herremar@unizar.es (M.H.); 2 Faculty of Pharmacy, University of Valencia, Burjassot 46100, Valencia, Spain; E-Mail: jesus.blesa@uv.es

**Keywords:** deoxynivalenol, bread making, wheat germ

## Abstract

Deoxynivalenol (DON, vomitoxin) is a type-B trichothecene mycotoxin produced by several field fungi such as *Fusarium graminearum* and *Fusarium culmorum* and known to have various toxic effects. This study investigated the effect of the bread making process on the stability of DON in common bread and wheat germ-enriched bread using naturally contaminated ingredients at the level of 560 µg/kg. The concentration of DON and its evolution during bread making were determined by immunoaffinity column cleanup followed by liquid chromatography with diode array detection (HPLC-DAD). During the bread making process, DON was reduced by 2.1% after fermentation and dropped by 7.1% after baking, reaching a maximum reduction of 19.8% in the crust as compared with a decrease of 5.6% in the crumb. The addition of 15% wheat germ to the dough did not affect DON stability during bread making, showing an apparent increase of 3.5% after fermentation and a reduction by 10.2% after baking.

## 1. Introduction

Wheat bread is a staple food prepared by baking a dough of flour and water usually leavened with yeast, which is widely consumed around the world [1]. In Spain, the mean consumption of bread accounts for 86 g/day [2]. Wheat germ is a component of wheat kernel with high nutritional value for the concentration of α-tocopherol (vitamin E), vitamins of group B, dietary fiber, polyunsaturated fats, proteins of high nutritive value, minerals and phytochemicals (*i.e.*, flavonoids). Consequently, wheat germ has been used as a flavoring ingredient in the manufacture of enriched breads available in the marketplace, increasing the nutritional value as well as extending shelf-life due to the natural content of organic acids and antifungal compounds such as lectin wheat germ agglutinin [3,4]. For all these reasons wheat germ and its derivatives are attractive and promising functional ingredients.

Deoxynivalenol (DON, vomitoxin) is a type-B trichothecene mycotoxin produced by several field fungi, including *Fusarium graminearum* and *Fusarium culmorum*, that cause a wide range of toxic effects in animal and humans [5,6]. Among the trichothecenes DON is the most frequently occurring toxin, and is found worldwide, particularly in cereal crops such as wheat and their products like flour, bread and germ [7,8,9]. To reduce the dietary exposure to DON, maximum limits have been set in flour (750 μg/kg) and bread (500 μg/kg) by the European legislation [10], and a temporary tolerable daily intake (TDI) of 1 μg/kg body weight was established.

The bread making process consists of three major stages: mixing, fermentation and baking. The fermentation and baking conditions vary considerably throughout the world, resulting in different effects on DON levels in final baked bread [11]. Bakery processing has been reported to reduce overall DON contamination [12,13,14,15], while others suggested that DON is highly stable during this process [16,17]. Similarly, Samar *et al.* [13] reported reductions in DON content during the fermentation phase, whereas Valle-Algarra *et al.* [14] did not observe any changes and Young *et al.* [18] even showed an increase of DON in the leavened products. These discrepancies may be due to several reasons such as the activity of baker’s yeast, which may produce a reduction of DON levels attributed to mycotoxin degradation or yeast absorption [13]. Then, the addition of wheat germ to the bread dough recipe may reduce the degradation rate of DON by affecting the baker’s yeast, as wheat germ is known to have natural antifungal compounds. In a recent review, it is concluded that the description of DON behavior during the bread making process is very difficult, since complex physico-chemical modifications occur during the process [19].

In summary, results of bread making studies on the stability of DON have been conflicting, and the effect of wheat germ addition on the mycotoxin level during bread making has not been studied. Therefore, the aims of the present work were to evaluate the stability of DON during bread making and to estimate the effect of wheat germ addition on the DON levels during the fermentation and baking phases of the bread making process.

## 2. Results and Discussion

The analytical method used for DON quantification in bread products, based on water extraction, immunoaffinity column cleanup and high performance liquid chromatography (HPLC) coupled with diode array (DAD) detection, was successfully validated down to 70 µg/kg. The method provided good recoveries for DON of 96.2%, and the study of intra-day precision in terms of repeatability obtained RSDr values of 4.5%, in accordance with the validation criteria [20].

**Figure 1:**
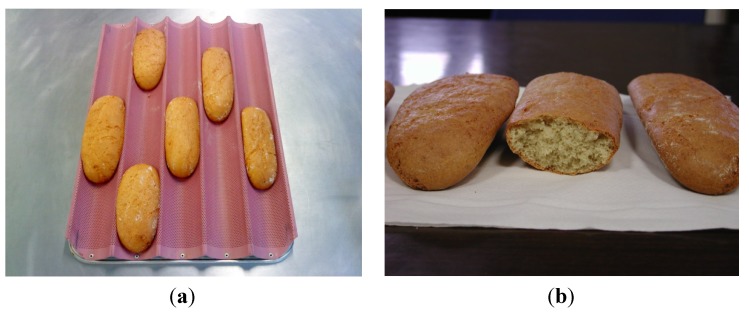
(**a**) Common bread and (**b**) enriched bread with 15% wheat germ.

Results of DON reduction during the different stages of bread making process are shown in Table 1. In common bread (Figure 1a), DON level in the staring material (560 µg/kg on a dry matter basis) was negligibly reduced by 2.1% during the fermentation step at 30 °C for 90 min and lowered by 7.1% after baking at 190 °C during 20 min. Evolution of DON levels in wheat germ-enriched bread (Figure 1b) showed an apparent increase by 3.5% after fermentation (from 560 to 580 µg/kg on a dry matter basis) followed by a reduction of 10.2% after baking. Therefore, the bread making process resulted in low reduction rates for DON in both bread types, which were non-significant as compared to the initial levels (*P* > 0.05). Consequently, the addition of 15% wheat germ did not exert any noticeable effect on the stability of DON during yeast fermentation and baking. 

Extra care should be exercised regarding the compliance with maximum limits established by Commission Regulation (EC) No. 1881/2006, depending whether analytical results are expressed on a fresh basis or on a dry matter basis. Actually, the DON level in common bread was 356 µg/kg on a fresh basis, below the maximum limit set at 500 µg/kg, but amounted to 520 µg/kg when calculated on a dry matter basis (moisture content was 31.5%) (Table 1).

The distribution pattern of DON in the crumb and the crust also evolved in a similar manner in common and enriched bread. Thus, DON reduction in finished common bread was 5.6% and 19.8% in the crumb and the crust, respectively, amounting to 7.1% and 17.9% in enriched bread. The greatest reductions of DON in both bread types were observed in the crust, the outer part of bread which supports the highest temperatures, although the differences were not significant (P > 0.05).

**Table 1:** Effect of fermentation and baking on the reduction of deoxynivalenol (DON) levels in common bread and enriched bread with 15% wheat germ.

Sample	Common bread		Wheat-germ enriched bread	
	DON µg/kg^a^	% loss	DON µg/kg	% loss
Dough before fermentation	560 ± 54	-	560 ± 62	-
Dough after fermentation	548 ± 56	2.1	580 ± 30	+3.5
Baked bread	520 ± 10	7.1	503 ± 25	10.2
Bread crumb	529 ± 74	5.6	520 ± 26	7.1
Bread crust	449 ± 45	19.8	460 ± 4	17.9

Note: ^a^: results expressed on a dry matter basis, as mean ± standard deviation (n = 2 assays).

According to the literature review, the main factors affecting the variability of the fate of DON during the bread making process include the preparation of the batter (ingredients and additives, mixing time), the fermentation step (yeast, incubation temperature and time), and the heating operation (oven-type, baking temperature and time). Some discrepancy of DON retention during baking bread may also result from uncertainty of the analytical method for DON. Thus, several studies have reported varying reductions in DON levels during bread making. Previous research showed DON overall reduction rates of 38%–44% [12], 48% [14], and 33%–58% [15]. On the other hand, other researchers reported that DON is highly stable during bread making [16,17]. These discrepancies may be due in large part to the analytical methods used, the concentration and source of toxin (natural *vs.* spiked), and the experimental conditions employed [21,22]. Thus, it has been reported that the ingredients used [23], the oven technology (commercial or home-made) [19,24] and the fermentation and baking conditions [14] influence on the reduction of DON level observed during bread making. On the other hand, Sugita-Konishi [17] reported that the DON level in flour was not reduced by bread making but that rather the biological toxicity was significantly reduced as determined by cytotoxicity bioassay.

Bakery processing has been reported to produce a thermal degradation of DON during bread making. For instance, the average reduction in DON concentration after baking (70 min at 195–235 °C) was 47.2% for bread baked in an industrial oven and 48.7% for bread baked in a log fire oven [25]. As reviewed by Kushiro [22], during baking or heating, DON is partially degraded to DON-related chemicals. Likewise, it is suggested that some DON reductions may be due to binding or the inability to extract the toxin from the matrix using current analytical techniques. Our results showed DON reductions up to 10.2% by baking at 190 °C for 20 min, which increased up to 19.8% in the crust that reached higher temperatures. Numanoglu *et al.* [26] indicated that the temperatures recorded in the crust and crumb of maize bread during baking were 100 °C and 150 °C, respectively, and thermal degradation of DON only initiated at 150 °C.

DON reduction during bread making may occur not only in the bakery due to thermal decomposition, but also during the fermentation step. Thus, yeast fermentation has been reported to produce a reduction of DON levels, which was attributed to mycotoxin degradation or yeast absorption. The fermentation stage during bread making produced DON reductions between 0% and 25% in dough fermented at 30 °C for 60 min, whereas there was a maximum 56% reduction when the dough was fermented at 50 °C [13]. In our study, yeast fermentation at 30 °C for 90 min produced minor changes of DON levels in the fermented dough, even an apparent increase of 3.5% in wheat germ-enriched bread. This is in agreement with Young *et al.* [18] who observed an increase in DON levels in yeast doughnuts, explained by the contamination of wheat with a DON precursor, which was possibly converted to DON by the active yeast. 

## 3. Experimental Section

### 3.1. Bread Making and Sampling

According to a typical baker recipe, two different types of bread were manufactured at the pilot plant of the Veterinary Faculty of Zaragoza (Spain): common bread and enriched bread with 15% of wheat germ. For each type of bread (common and enriched) there were two bread making assays carried out in different times. Each assay consisted of 1.0 kg of dough that yielded 6 bread pieces of approximately 150 g. Therefore, for each bread type there were a total of two analytical samples (*n* = 2 assays) at each processing step: dough before fermentation (50 g each), dough after fermentation (50 g each), baked bread (50 g pooled from two bread pieces), bread crumb (50 g pooled) and bread crust (50 g pooled). For statistical purposes, each result in Table 1 is the mean ± standard deviation (*n* = 2 assays). The bread making process showed good repeatability between the two assays as indicated by adequate values of relative standard deviation (%RSD) calculated from Table 1. The mean %RSD was 7.3% and individual RSD values depending on sample type ranged from 0.9% to 14%.

The bread formulas were as follows: (i) common bread made with 1000 g wheat flour, 550 mL tap water, 16 g sodium chloride, and 40 g of commercial baker’s yeast (*Saccharomyces cerevisiae*), and (ii) enriched bread made with 850 g wheat flour, 150 g wheat germ, 550 mL tap water, 16 g sodium chloride, and 40 g of commercial baker’s yeast. A continuous high-speed mixer was used to prepare the batter by adding 450 mL pre-warmed water (37–40 °C) and mixing for 3 min, followed by the addition of baker’s yeast dissolved in 100 mL pre-warmed water and mixing for another 8 min. Dough was settled at room temperature for 15 min, and then fermentation was carried out during 90 min in a camera at 30 °C and 80% relative humidity. Finally, the raised dough was baked in an oven at 190 °C for 20 min to obtain the bread. 

DON levels were determined at each step of the bread making: dough before fermentation, dough after fermentation, and baked bread. For each bread several slices were cut and samples were taken from the crumb and the crust. The wheat flour and wheat germ used as main ingredients were naturally contaminated with DON and produced a concentration of 560 µg DON/kg dry matter in the starting material (dough before fermentation).

### 3.2. Reagents and Apparatus for DON Analysis

HPLC grade acetonitrile and methanol were purchased from Lab-Scan (Dublin, Ireland). Ultrapure water was obtained from a Milli-Q Plus apparatus from Millipore (Milford, MA, USA). The immunoaffinity columns DonStar™ were supplied by Romer Labs (Union, MO, USA). Deoxynivalenol standard solution at 100 µg/mL in acetonitrile was provided by Sigma (St. Louis, MO, USA) and stored at −21 °C. Reagents for phosphate-buffered saline solution (PBS) were obtained from Panreac (Barcelona, Spain).

The LC system consisted of an Agilent Technologies (Santa Clara, CA, USA) 1100 high performance liquid chromatograph coupled to an Agilent diode array detector (DAD) at 220 nm for the determination of DON. The LC column was Ace 5 C18, 250 mm × 4.6 mm, 5 μm particle size (Advanced Chromatography Technologies, Aberdeen, UK). The mobile phase consisted of a mixture of water/acetonitrile/methanol (90:5:5, v/v/v) at a flow rate of 1.0 mL/min.

### 3.3. Analysis of DON in Dough and Bread Samples

For the determination of DON in dough and bread samples, five grams were extracted with 40 mL of Milli-Q water using an Ultraturrax homogenizer for 3 min. After the extraction, the solution was filtered with Whatman #4 filter paper, and the extract collected for further cleanup by DonStar™ immunoaffinity columns according to the manufacturer’s instructions. Briefly, 2 mL of the filtered extract were passed through the column at a flow-rate of 1 drop/second, followed by a washing with 5 mL PBS pH 7. DON was then eluted with 3 × 0.5 mL methanol and collected in a clean vial. The eluted extract was evaporated to dryness under nitrogen stream at 50 °C and redissolved with 400 µL of the HPLC mobile phase. One hundred µL was injected into the LC-DAD system by full loop injection system. Quantification of DON was performed by measuring peak areas at DON retention time, and comparing them with the relevant calibration curve. To facilitate the comparison of the DON levels in the different samples taken during the bread making process, results were expressed on a dry matter basis. For this purpose, a sample of 5 g was heated in an oven at 130 °C for 2 h. After cooling, the moisture content was determined by weight loss and the mycotoxin content expressed on a dry matter basis according to the formula:

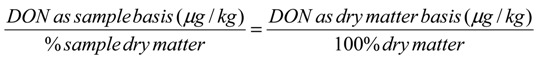
(1)


Average moisture contents were 41.8% for dough before fermentation, 41.7% dough after fermentation, 31.5% bread, 40.7% crumb, and 16.4% crust.

### 3.4. Statistical Analyses

Results from mycotoxin analyses were subjected to descriptive and comparative statistics according to Sachs [27]. DON levels determined before and after each processing step of the bread baking assays and between different sample types (bread crumb, crust) were statistically evaluated by the one-way analysis of variance (ANOVA at *P* = 0.05) procedure using the statistical software StatView SE + Graphics (Abacus Concepts, Berkeley, CA, USA). Fisher PLSD test was used when significant differences were found among means.

## 4. Conclusions

Mycotoxins are considered to be very stable molecules but because of their toxic effects, information about their stability in thermal processes and potential inactivation procedures is needed. This study concluded that DON was stable during the bread making process and remained stable in the enriched bread with 15% wheat germ. The fermentation step (30 °C for 90 min) and the oven baking (190 °C for 20 min) resulted in non-significant losses of DON from the initial dough. Quite different results concerning the fate of DON during bread making have been reported in the literature to date. The discrepancies between the findings reported in these different studies may be due in large part to the analytical methods employed, the differences in the experimental conditions employed and the concentration and source of toxin.
